# A genetic analysis of a Spanish population with early onset Parkinson’s disease

**DOI:** 10.1371/journal.pone.0238098

**Published:** 2020-09-01

**Authors:** Tejera-Parrado Cristina, Mir Pablo, Periñán María Teresa, Vela-Desojo Lydia, Abreu-Rodríguez Irene, Alonso-Cánovas Araceli, Bernal-Bernal Inmaculada, Bonilla-Toribio Marta, Buiza-Rueda Dolores, Catalán-Alonso María José, García-Ramos Rocío, García-Ruiz Pedro José, Huertas-Fernández Ismael, Jesús Silvia, Miguel A-Espinosa Labrador, López-Manzanares Lydia, Martínez-Castrillo Juan Carlos, Ignacio J. Posada, Rojo-Sebastián Ana, Ruiz-Huete Cristina, Del Val Javier, Pilar Gómez-Garre

**Affiliations:** 1 Unidad de Trastornos del Movimiento, Servicio de Neurología y Neurofisiología, Instituto de Biomedicina de Sevilla (IBiS), Hospital Universitario Virgen del Rocío/CSIC/Universidad de Sevilla, Seville, Spain; 2 Centro de Investigación Biomédica en Red sobre Enfermedades Neurodegenerativas (CIBERNED), Madrid, Spain; 3 Servicio de Neurología, Hospital Fundación Alcorcón, Madrid, Spain; 4 Servicio de Neurología, Hospital Ramón y Cajal, Madrid, Spain; 5 Servicio de Neurología, Hospital Clínico San Carlos, Madrid, Spain; 6 Servicio de Neurología, Fundación Jiménez Díaz, Madrid, Spain; 7 Servicio de Neurología, Hospital La Princesa, Madrid, Spain; 8 Servicio de Neurología, Hospital Universitario 12 de Octubre, Madrid, Spain; 9 Servicio de Neurología, Hospital Príncipe de Asturias, Madrid, Spain; 10 Servicio de Neurología, Clínica Nuestra Señora del Rosario, Madrid, Spain; Oslo Universitetssykehus, NORWAY

## Abstract

**Introduction:**

Both recessive and dominant genetic forms of Parkinson’s disease have been described. The aim of this study was to assess the contribution of several genes to the pathophysiology of early onset Parkinson’s disease in a cohort from central Spain.

**Methods/patients:**

We analyzed a cohort of 117 unrelated patients with early onset Parkinson’s disease using a pipeline, based on a combination of a next-generation sequencing panel of 17 genes previously related with Parkinson’s disease and other Parkinsonisms and CNV screening.

**Results:**

Twenty-six patients (22.22%) carried likely pathogenic variants in *PARK2*, *LRRK2*, *PINK1*, or *GBA*. The gene most frequently mutated was *PARK2*, and p.Asn52Metfs*29 was the most common variation in this gene. Pathogenic variants were not observed in genes *SNCA*, *FBXO7*, *PARK7*, *HTRA2*, *DNAJC6*, *PLA2G6*, and *UCHL1*. Co-occurrence of pathogenic variants involving two genes was observed in *ATP13A2* and *PARK2* genes, as well as *LRRK2* and *GIGYF2* genes.

**Conclusions:**

Our results contribute to the understanding of the genetic architecture associated with early onset Parkinson’s disease, showing both *PARK2* and *LRRK2* play an important role in Spanish Parkinson’s disease patients. Rare variants in *ATP13A2* and *GIGYF2* may contribute to PD risk. However, a large proportion of genetic components remains unknown. This study might contribute to genetic diagnosis and counseling for families with early onset Parkinson’s disease.

## Introduction

Parkinson’s disease (PD) is the second most common neurodegenerative disorder characterized by the loss of dopaminergic neurons in the *substantia nigra*. It is a chronic and progressive disorder of multifactorial etiology, in which causative and susceptibility genetic factors are involved. Emerging evidence has provided support for the hypothesis that PD is the result of complex interactions among genetic abnormalities, environmental toxins, mitochondrial dysfunction, and other cellular processes.

Since Polymeropoulos et al. identified a causative variant in gene encoding Alpha synuclein (*SNCA*) [1], many efforts have been made to identify genes involved in the development of PD. Mutations in *SNCA*, Leucine-rich repeat kinase 2 (*LRRK2*), and Vacuolar Protein Sorting 35 (*VPS35*) genes have been linked to autosomal dominant forms of PD (ADPD) [2, 3]. In fact, the mutations in *LRRK2* are the most common cause of ADPD. In addition, the Eukaryotic Translation Initiation Factor 4 Gamma 1 (*EIF4G1*), was initially related to ADPD, but subsequent studies have failed to replicate this association [4]. Furthermore, genes related to autosomal recessive forms of PD (ARPD) have also been discovered. Thus, the genes coding for Parkin (*PARK2*), PTEN-induced kinase 1 (*PINK1*), and the Protein deglycase DJ1 (*PARK7*) have been related to typical early-onset PD (EOPD; age at onset <50 years old). Other genes, such as those coding for the ATPase Cation Transporting 13A2 (*ATP13A2*), the Phospholipase A2 Group VI (*PLA2G6*), the F-Box Protein 7 (*FBXO7*), HSP40 Auxilin (*DNAJC6*) and the Synaptojanin-1 (*SYNJ1*), have been related to atypical PD with juvenile onset (age at onset <35 years old) [4]. On the other hand, the gene *GIGYF2* (encoding Grb10-Interacting GYF Protein 2) has been described as responsible for typical autosomal dominant PD [5]. Its pathogenic contribution to PD is still not clear but it has been suggested that some variations are risk factors for PD in Caucasians [6].

Nevertheless, monogenic mutations are not a very common cause of PD. Indeed, risk variants with a moderate effect size are more usual.

Variations in genes such as *GBA* (encoding glucocerebrosidase) or *SMPD1* (encoding sphingomyelin phosphodiesterase 1) constitute risk factors for PD. In fact, variants in *GBA* have been proposed as the most important risk factor for idiopathic PD [7].

To date, genome wide association studies (GWAS) have allowed identification of several PD risk loci [8]. However, a large proportion of genetic heritability remains unknown. Nowadays, next generation sequencing (NGS) of whole genome (WGS) or just the exome (WES) are expected to contribute to elucidating the missing heritability through the identification of new risk variants related to PD.

Since genetic background has a higher impact on PD with early disease onset, the aim of this study was to assess the contribution of seven genes previously related to PD, and unconfirmed genes, to the pathophysiology of the EOPD in a cohort of patients from central Spain. For that purpose a combination of NGS-based targeted sequencing and Multiplex Ligation-Dependent Probe Amplification (MLPA) were applied.

## Patients and methods

### Subjects and clinical assessments

We included 117 unrelated EOPD (age at onset younger than 50 years old) patients. Among them, thirty-three reported a family history of PD. The demographic characteristics of the participants are summarized in Table 1. Patients were Caucasian and recruited from the Neurology outpatient clinic at different hospitals in the Comunidad Autónoma de Madrid (Spain) and clinically evaluated. An extensive set of clinical features was obtained. PD was diagnosed by Movement Disorders neurologists according to the United Kingdom Parkinson’s Disease Society Brain Bank criteria [9]. After clinical diagnosis, peripheral blood samples were collected from each subject.

**Table 1:** Demographic characteristics of PD patients.

		Sex (M/F)	Age ± SD (y)	AO ± SD (y)	N
**EOPD**	**Total**	64/53	51.7 ± 9	40.2 ± 5.3	117
	**Familial PD**	17/16	52.2 ± 9.2	39.2 ± 5.5	33
	**Sporadic PD**	47/37	51.5 ± 9	40.8 ± 5	84

EOPD: early-onset Parkinson's disease (age at onset <50 years old); PD: Parkinson's disease; M: males; F: females; y: years; AO: age at onset; SD: standard deviation; N: number of samples.

### Ethics statements

The study was approved by the CEIs (Comités de Ética en Investigación) from all participating centers (S1 Methods), and it was conducted according to the principles expressed in the Helsinki Declaration. Each individual who participated in the study signed a written informed consent form prior to blood withdrawal.

### Genetic analysis

#### DNA isolation

Genomic DNA was isolated from peripheral blood samples from each subject according to established protocols, by manual and automated commercial procedures (Roche Applied Science, Indianapolis, IN, USA). The quantity and purity of the DNA were determined by Qubit 3.0 fluorometer (Invitrogen) and NanoDrop2000 spectrophotometer (Thermo Fisher Scientific, Florida, USA), respectively.

#### Targeted gene enrichment and next-generation sequencing (NGS)

Targeted re-sequencing was performed using a customized Haloplex Target Enrichment Panel, which was designed using Agilent's online Sure Design tool, following the manufacturer’s protocol (Agilent Technologies, Inc. Santa Clara, CA). This customized panel covered all coding exons, exon-intron boundary regions, and 3' and 5' untranslated regions of 17 selected genes. These genes were those related to dominant and recessive forms of PD, as well as risk genes, and unconfirmed genes: *SNCA*, *LRRK2*, *PINK1*, *PARK2*, *ATP13A2*, *FBXO7*, *VPS35*, *DJ1*, *PLA2G6*, *EIF4G1*, *DNAJC6*, *HTRA2*, *SMPD1*, *SYNJ1*, *UCHL1*, *GIGYF2*, and *GBA*.

Sample preparation was carried out according to the manufacturer’s protocol. The concentration of the enriched and amplified samples was determined using a Bioanalyzer High Sensitivity chip (Agilent Technologies). Then, samples were pooled in equimolar amounts and sequenced at the Illumina NextSeq platform (Illumina Inc., San Diego, CA, USA).

#### Bioinformatics analysis for NGS results

**Fig 1:**
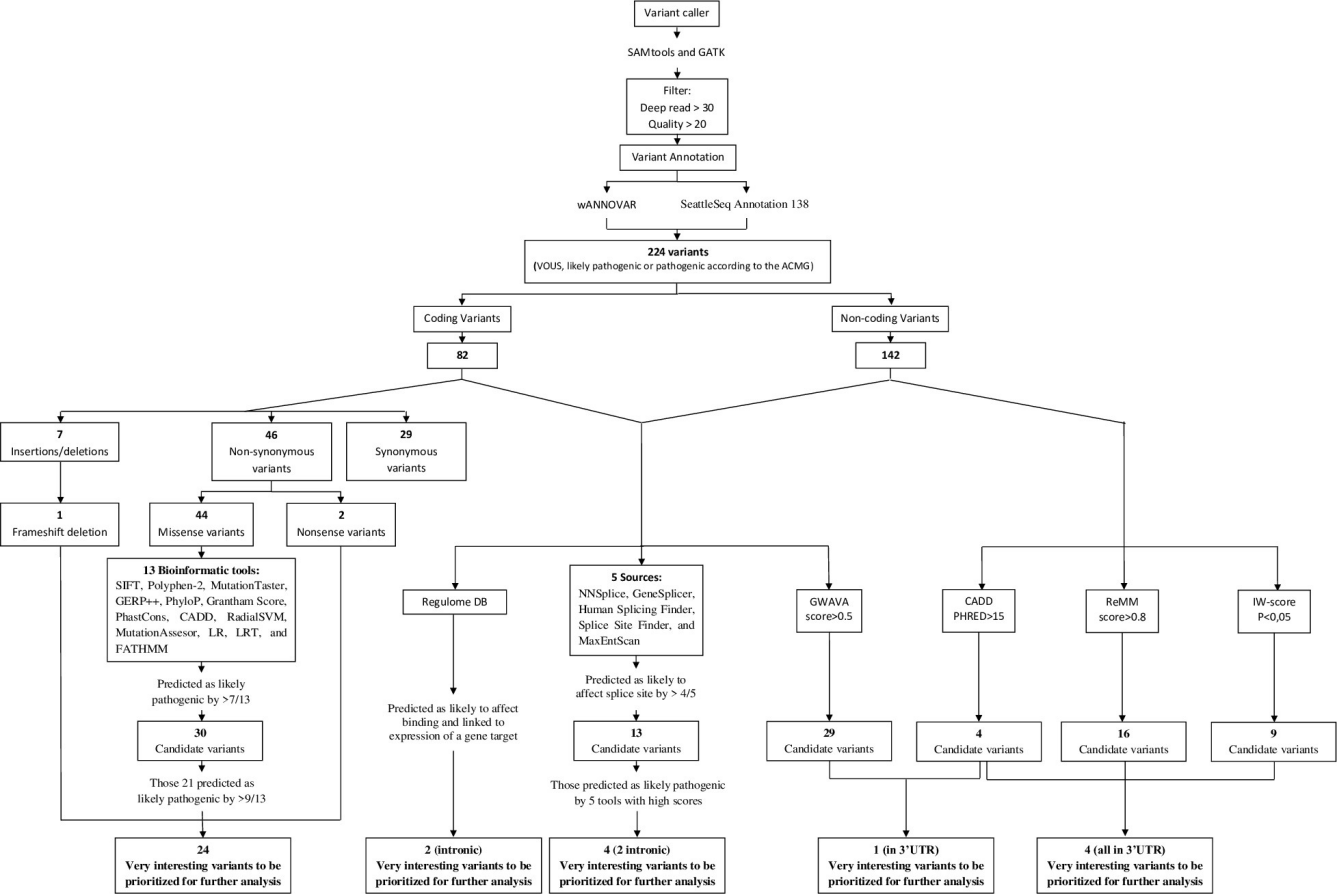
Flowchart of sequencing data analysis. Horizontal boxes represent steps in the workflow. *In silico* analysis were carried out in 224 SNVs. SIFT: Sorting Intolerant From Tolerant. Polyphen-2: Polymorphism Phenotyping 2. GERP: Genomic Evolutionary Rate Profiling. CADD: Combined Annotation Dependent Depletion. RadialSVM: Radial Support Vector Machine. LR: Logistic Regresion. LRT: Likelihood Ratio Test. FATHMM: Functional Analysis Through Hidden Markov Models. GWAVA: Genome Wide Annotation of Variants. ReMM: Regulatory Mendelian Mutation. IW-score: Integrative Weighted score (with an associated p-value<0.5).

Data analysis and prioritization of variants were performed using the workflow summarized in Fig 1.

Briefly, sequenced reads quality were examined by FastQC (Babraham Bioinformatics, Cambridge, UK) and poor quality bases were trimmed by Cutadapt v1.9.1. The reference genome was downloaded from NCBI, version GRCh37/hg19. The Burrows-Wheeler Aligner v0.7.12 was used to align the remaining clean reads. All variants, single nucleotide polymorphisms (SNP) and small indels, were detected using SAMtools v1.2. Variants were filtered by Bcftools v1.2 and GATK v4.0, and only those with depth read over 30 and quality over 20 remained. Subsequently, variants were annotated using SeattleSeq Annotation 138 (http://snp.gs.washington.edu/SeattleSeqAnnotation138/) and wANNOVAR (http://wannovar.wglab.org/index.php). The variants where the fraction of non-reference reads was <0.3 were rejected. In addition, a scoring and priorization analysis of deleterious alleles were performed using the software Expert Variant Interpreter (eVAI), from enGenome (www.engenome.com/product) and variants were categorized according to the international guidelines of American College of Medical Genetics and Genomics (ACMG) [10].

For the selection of candidate variants we followed the combination of several criteria (S1 Methods). Missense variants were analyzed with a total of 13 bioinformatic tools. Furthermore, all variants were analyzed using the Batch version of the annotation software package Alamut (Interactive Biosoftware, Rouen, France) for splicing predictions. A threshold was set for each tool above, in which the variant was classified as damaging (S1 Table). The impact was interpreted in relation to all known isoforms of each gene. To restrict the analysis, allele frequencies on population databases were also taken into account.

#### Sanger resequencing

Filtered variants predicted as pathogenic were validated by Sanger sequencing. Exons containing each selected variant were amplified using standard PCR protocol. Amplicons were sequenced on both strands using the BigDye terminator cycle sequencing kit (Applied Biosystem, Foster City, CA, USA) and, subsequently, they were resolved on an ABI3500 genetic analyzer and analyzed by software Variant Reporter v1.1 (Applied Biosystem).

In order to avoid the amplification of the neighboring pseudogene, *GBA* was first amplified in four large fragments that only and specifically amplified the functional gene but not the nearby pseudogene, specific exons were then amplified and sequenced.

#### Copy Number Variation (CVN) analysis

Multiplex ligation-Dependent Probe Amplification (MLPA) was carried out using two commercially available SALSA MLPA kits (P051 and P052; MRC-Holland, Amsterdam, The Netherlands), following the manufacturer’s recommendations. Data were analyzed with the Coffalyser.Net software (MRC-Holland). Analysis of NGS data for CNV identification was performed with VisCap software. Losses were defined by a maximum log2 ratio of -0.55 and a minimum log2 ratio of 0.4 for gains [11].

## Results

**Fig 2:**
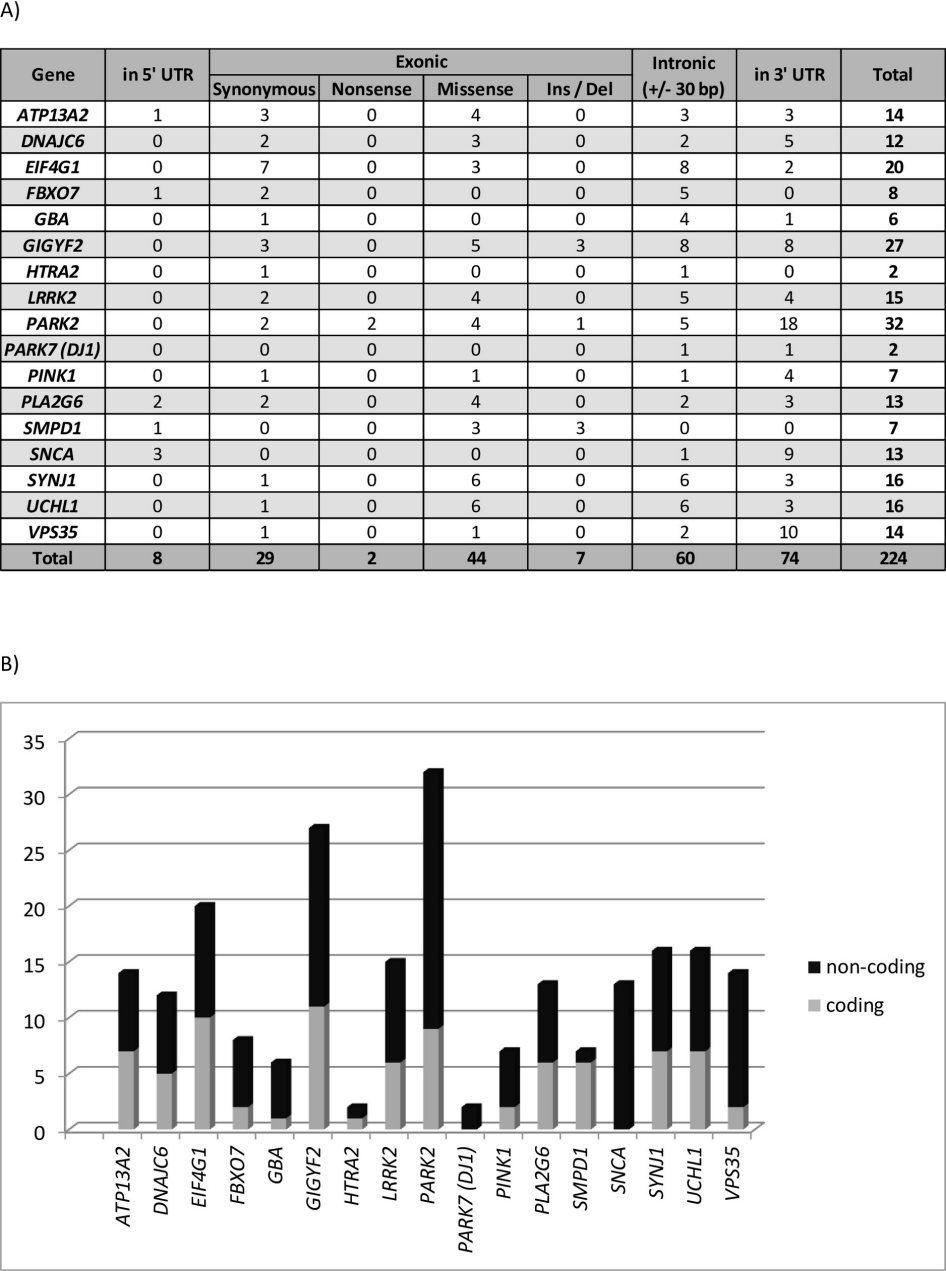
Number and distribution of detected variants in our population using a 17-gene sequencing panel. Variants in all 17 genes were evaluated and only those described *a priori* as a variant of unknown significance, likely pathogenic or pathogenic, according to ACMG, are shown. Genes with variants and the variant distribution are displayed (A), and the proportions of coding and non-coding variants are shown for all genes (B).

In this study, a total of 224 variants (categorized as variant of unknown significance, likely pathogenic or pathogenic according to the ACMG) were detected by bioinformatics analysis of NGS data, with 82 coding variants and 142 non-coding variants (Fig 2). Subsequently, all of them were further characterized using several algorithms, following the aforementioned analysis, to analyze their putative functional impact (Fig 1). In addition, in CNV analysis, only deletion affecting to exons of *PARK2* were detected.

### Identification of the most interesting variants

Firstly, 32 variants, that included the nonsense variants and those missense variants, were kept. These variants were predicted as likely pathogenic at 54% (7 out of 13) of the bioinformatics tools (S2 Table). However, due to prediction tools, in general, have a tendency of over-predicting deleterious impacts, only those variants with >69% *in silico* predictions in favor of pathogenicity, and/or described as pathogenic in ClinVar were considered as very interesting variants to be prioritized for further analysis (S3 Table).

Three variants, all of them in *PARK2*, were directly assigned as loss-of-function because they disrupt the protein: two of them were nonsense variants, and one was a deletion of one nucleotide resulting in a frameshift (S3 Table).

In addition, four different deletions affecting to entire exons (all of the gene *PARK2*) were identified: three single-exon deletions and one affecting four consecutive exons (S3 Table).

Deeper analysis of intronic nsertions or deletions of a few bases and those variations located in the untranslated regions were considered for further analysis.

Thirteen variants were predicted bioinformatically to have a putative effect on splicing (S4 Table). Of them, four variants (rs560897844, rs112019125, rs148944108, and rs41286476) were predicted as likely pathogenic by all tools with high scores, and they presented allele frequencies higher than those in databases (S4 Table).

Two intronic variants had a score of 1f (those that are known eQTLs for genes and have TF binding or a DNase peak) in the RegulomeDB scoring system: rs6437074 (in *GIGYF2*) and rs2298298 (in *PINK1*). However, their allele frequencies in our population were very high and similar to those previously described in databases, thus they were excluded.

Lastly, we kept only those variants that were in a heterozygous state in dominant genes, and in homozygous or compound heterozygous states for recessive ones.

**Fig 3:**
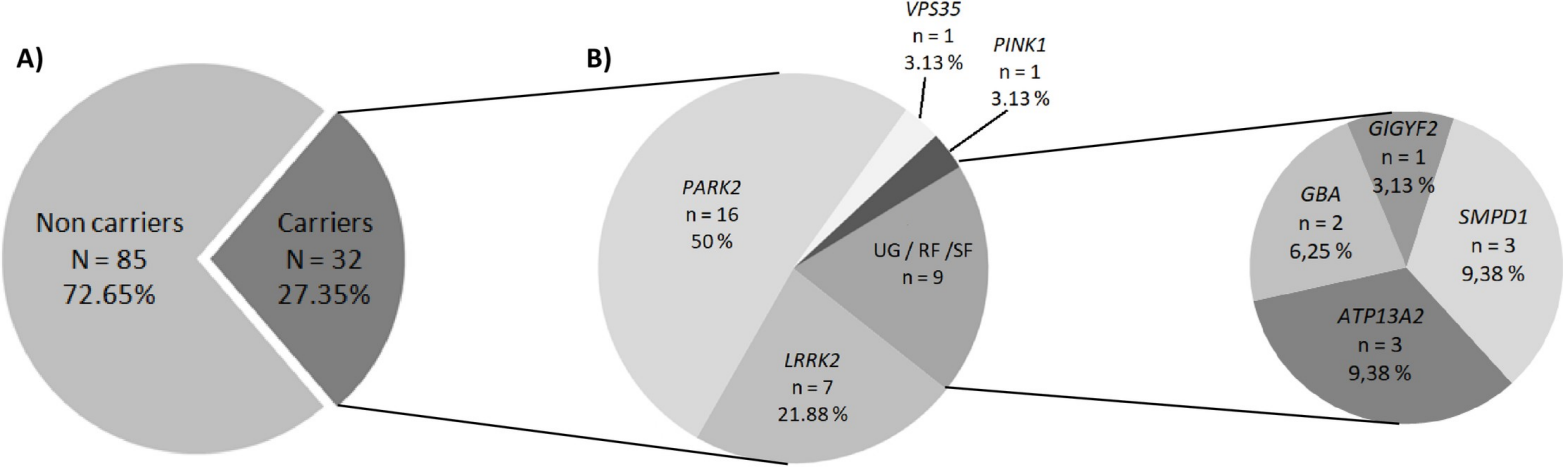
Pie plot showing the distribution and frequencies of likely pathogenic variants found. In our population, we identified 32 patients carrying likely pathogenic variants (A), of which more than a half presented variations in PARK2 (50%) or LRRK2 (21.88%) (B). UG: Unconfirmed genes. RF: Risk factor. SF: Susceptibility factor. N: number of subjects. n: number of carriers subjects with pathogenic variations in each gene.

In this way, we identified 32 of the 117 studied patients (27.35%) to be carrying potentially pathogenic variants across 8 genes (Fig 3). Seven patients carried likely pathogenic variations, in *LRRK2*, 16 in *PARK2*, 1 in *VPS35*, and 1 in *PINK1*. In addition, 9 patients carried likely pathogenic variations in the risk factors: 3 in ATP13A2, 3 in *SMPD1*, 2 in *GBA*, and 1 in *GIGYF2* (Table 2).

**Table 2:** Patients carrying likely pathogenic variants. Genotype-phenotype correlations.

Patient	Gene	Variation (HGVS)		Zigosity	VAF	Coverage (Alt/Ref)	Sex	Age at onset	Family history	Dopaminergic complications (AAOS)			Non-motor symptoms		
		CDS	AA							MF	DK	N-MF	Depression	CI	VH
**EP-1**	*ATP13A2*	c.649G>A	p.Gly217Ser	Het	0.467	353 (165/188)	F	48	No	No	No	No	No	No	No
**EP-2**	*ATP13A2*	c.2234G>A	p.Arg745His	Het	0.466	562 (262/300)	F	45	Yes	Yes (8)	Yes (8)	No	No	No	No
**EP-5**	*SMPD1*	c.441G>A	p.Val147Val	Het	0.469	1453 (681/772)	M	45	No	Yes (4)	Yes (5)	No	Yes	No	No
**EP-6**	*SMPD1*	c.441G>A	p.Val147Val	Het	0.497	1264 (628/636)	M	49	No	Yes (4)	Yes (1)	Yes	No	No	No
**EP-7**	*VPS35*	c.1960G>T	p.Ala654Ser	Het	0.323	563 (182/381)	F	45	-	Yes	Yes	Yes	yes	No	Yes
**EP-8**	*GBA*	c.1040T>C	p.Ile347Thr	Het	0.490	1117 (547/570)	M	36	Yes	No	No	No	No	No	No
**EP-9**	*GBA*	c.1208G>A	p.Ser403Asn	Het	0.462	764 (353/411)	M	40	Yes	Yes (3)	Yes (4)	Yes (2)	No	No	No
**EP-10**	*PINK1*	c.1040T>C	p.Leu347Pro	Hom	0.981	1776 (1743/33)	M	25	No	No	Yes	No	No	No	No
**EP-11**	*PARK2*	c.79A>T	p.Lys27Stop	Hom	0.997	567 (565/2)	F	41	No	No	No	No	No	No	No
**EP-12**	*PARK2*	c.155delA	p.Asn52Metfs	Hom	0.985	1092 (1076/16)	M	35	Yes	Yes	No	No	No	No	No
**EP-13**	*PARK2*	c.155delA	p.Asn52Metfs	Hom	1	450 (450/0)	F	32	No	Yes	Yes	No	No	No	No
**EP-14**	*PARK2*	c.155delA	p.Asn52Metfs	Hom	0.985	391 (385/6)	F	37	-	Yes	Yes	Yes	No	Yes	No
**EP-15**	*PARK2*	c.155delA	p.Asn52Metfs	Hom	0.983	591 (581/10)	F	31	No	No	No	No	Yes	Yes	No
**EP-16**	*PARK2*	c.155delA	p.Asn52Metfs	Hom	0.998	875 (873/2)	F	42	No	Yes	No	No	No	No	No
**EP-17**	*PARK2*	c.155delA	p.Asn52Metfs	Hom	0.997	387 (386/1)	F	24	Yes	No	No	No	Yes	No	No
	*ATP13A2*	c.2762+21T>C	-	Het	0.531	443 (235/208)									
**EP-18**	*PARK2*	c.155delA	p.Asn52Metfs	Het	0.336	935 (314/621)	F	37	No	Yes	Yes	Yes	Yes	Yes	Yes
	*PARK2*	Del ex 7	-	Het	-	-									
**EP-19**	*PARK2*	c.155delA	p.Asn52Metfs	Het	0.318	1407 (447/960)	F	37	No	Yes	Yes	Yes	Yes	No	No
	*PARK2*	Del ex 7	-	Het	-	-									
**EP-20**	*PARK2*	c.155delA	p.Asn52Metfs	Het	0.348	811 (282/529)	F	28	No	Yes	Yes	Yes	No	No	No
	*PARK2*	c.719C>T	p.Thr240Met	Het	0.413	1532 (632/900)									
**EP-21**	*PARK2*	c.766C>T	p.Arg256Cys	Het	0.525	1959 (1029/930)	M	44	No	No	No	No	No	No	No
	*PARK2*	Del ex 7	-	Het	-	-									
**EP-22**	*PARK2*	c.1205G>A	p.Arg402His	Het	0.512	1256 (643/613)	M	40	No	Yes (12)	Yes (13)	Yes (10)	No	No	No
	*PARK2*	Del ex 7	-	Het	-	-									
**EP-23**	*PARK2*	Del ex 3	-	Hom	-	-	F	33	Yes	Yes	Yes	Yes	Yes	No	No
**EP-24**	*PARK2*	Del ex 3-4-5-6	-	Hom	-	-	M	34	Yes	Yes	Yes	No	No	No	No
**EP-25**	*PARK2*	Del ex 3-4-5-6	-	Hom	-	-	F	47	Yes	Yes (9)	Yes (7)	-	-	-	-
**EP-26**	*PARK2*	c.1334G>A	p.Trp445Stop	Het	0.520	321 (167/154)	F	34	Yes	No	No	No	No	Yes	No
	*PARK2*	Del ex 3-4-5-6	-	Het	-	-									
**EP-27**	*LRRK2*	c.6055G>A	p.Gly2019Ser	Het	0.442	981 (434/547)	M	29	No	Yes (3)	Yes (4)	No	No	No	No
**EP-28**	*LRRK2*	c.6055G>A	p.Gly2019Ser	Het	0.393	1011 (397/614)	M	40	Yes	Yes (12)	No	No	No	No	No
**EP-29**	*LRRK2*	c.6055G>A	p.Gly2019Ser	Het	0.442	1025 (453/572)	F	39	No	Yes (4)	Yes (5)	-	No	No	No
**EP-30**	*LRRK2*	c.6055G>A	p.Gly2019Ser	Het	0.369	1154 (426/728)	F	42	No	Yes (1)	Yes (1)	-	No	No	No
**EP-31**	*LRRK2*	c.6055G>A	p.Gly2019Ser	Het	0.419	1046 (438/608)	F	40	No	Yes (2)	Yes (2)	Yes (2)	No	No	No
**EP-32**	*LRRK2*	c.4321C>T	p.Arg1441Cys	Het	0.524	708 (371/337)	M	45	No	Yes (4)	Yes (5)	Yes (4)	No	No	No
**EP-33**	*LRRK2*	c.4536+3A>G	-	Het	0.460803	523 (241/282)	M	45	Yes	Yes	Yes	-	No	Yes	No
	*GIGYF2*	c.3673C>T	p.Arg1225Cys	Het	0.500606	825 (413/412)									
**EP-34**	*SMPD1*	c.1132C>T	p.Arg378Cys	Het	0.500808	1857 (930/927)	F	46	No	No	No	No	Yes	Yes	No

HGVS: Human Genome Variation Society (Variants have been described using HGVS nomenclature). CDS: Coding sequence. AA: Amino acid. Het: Heterozygous. Hom: Homozygous. VAF: Variant allele frequency. Alt: Alternate allele. Ref: Reference allele. M: Male. F: Female. MF: Motor fluctuations. NMF: Non-motor fluctuations. DK: Dyskinesia. AAOS: Age at onset of symptom. CI: Cognitive impairment. VH: Visual hallucinations.

### Findings in PD-associated genes

Twenty-six patients carried likely pathogenic variants in *PARK2*, *LRRK2*, *PINK1*, or *GBA*.

*PARK2* was the gene most frequently mutated (Fig 3; Table 2). Therefore, 16 patients presented pathogenic variants in *PARK2*, 8 of them carried deletions of entire exons of *PARK2*. The most often pathogenic variant in *PARK2* was a previously described frameshift variation (p.Asn52Metfs*29). To the best of our knowledge, all carriers for this variation are unrelated patients.

Seven patients carried previously described pathogenic variations in *LRRK2*. Of them, 5 patients carried the variation p.Gly2019Ser, 1 patient carried the variation p.Arg1441Cys, and 1 patient carried the intronic variation c.4536+3A>G.

No significant difference (*P* = 0.205) was shown between age at onset of patients with pathogenic variants in *LRRK2* (age at onset 40.1±5.6 years old) and those with pathogenic variants in *PARK2* (age at onset 36.7±5.7 years old).

We found one patient carrying a homozygous pathogenic variant in *PINK1* (p.Leu347Pro) and another patient with a heterozygous pathogenic variant in *VPS35* (p.Ala654Ser). Therefore, variations in these genes are a rare cause of disease in our population.

Finally, two patients carried two non-previously described likely pathogenic variants in *GBA* (p.Ile347Thr and p.Ser403Asn).

No pathogenic variant was observed in *SNCA*, *and PARK7*, illustrating their rarity in our population.

### Findings in other genes

A total of 6 variants in *ATP13A2* (p.Gly217Ser, p.Arg745His; and c.2762+21T>C), *GIGYF2* (p.Arg1225Cys), and *SMPD1* (p.Val147Val and p.Arg378Cys), were found as putatively contributing to PD (Table 2).

Pathogenic variants were not observed in *EIF4G1*, SYNJ1, *FBXO7*, *DNAJC6*, *PLA2G6*, *HTRA2*, and *UCHL1*.

## Discussion

In this study, a pipeline, based on a combination of a next-generation sequencing panel of 17 genes and MLPA, has been applied to detect PD-related variations in a Caucasian population with EOPD from Central Spain. Twenty-seven patients out of 117 (23.08%) showed likely pathogenic variants in known PD-associated genes.

Pathogenic variants in the *PARK2* gene were the most common genetic cause of early-onset ARPD, with a variable prevalence in previous studies in EOPD [12–14]. Therefore, *PARK2* variants were the most frequently identified explanation for PD in our cohort also (13.68%; 16 patients out of 117); with the most frequent pathogenic variant (6.4%; 15/234) being a previously described frameshift variation (p.Asn52Metfs*29) [15]. This result is different in comparison to a study with PD patients from northern Spain, which described this variation, in homozygous, in one patient from a population of 72 EOPD patients (1.4%; 2/144) [16]. However, in another previous study, that variation was found in 2 patients (in a homozygous and heterozygous state) from a 26 EOPD patient cohort (5.8%; 3/52) from southern Spain [17], similar to what it was obtained in our population. Those results suggest that, although p.Asn52Metfs*29 is geographically widespread, it is less prevalent in the Basque Country and it could be associated with a founder effect and subsequent dispersion of the variation.

Interestingly, one patient (EP-17) to be carrying the pathogenic variation p.Asn52Metfs*29 in *PARK2* in a homozygous state, also carried a variation in *ATP13A2* (c.2762+21T>C), which was predicted by the bioinformatic tools as affecting splicing due the apparition of a stronger cryptic 5' donor site. This patient had an age at onset that was younger than the other carriers of the same variation p.Asn52Metfs*29, suggesting a cumulative effect between both genes on age at onset. The *ATP13A2* gene has been found mutated in some types of early-onset Parkinsonism [18]. Although the pathogenicity of single heterozygous variations is a matter of debate, the reduction of functional ATP13A2 has been related to the development of PD as a risk factor. Moreover, it has been suggested that dopaminergic neurons expressing higher ATP13A2 protein levels are less susceptible to cell death [19]. Given the rarity of *ATP13A2* pathogenic variations, confirmation of this point will require sequencing of much larger sample sizes.

On the other hand, to date, pathogenic variations in *LRRK2* (a multi-domain protein with both GTPase and kinase functionality) are the most common genetic determinant of ADPD. The most frequent pathogenic variation described is p.Gly2019Ser, although its prevalence varies considerably [17, 20, 21]. We found this variation in 5 patients, which represents the 4.27% of the 117 studied samples. This frequency is consistent with the prevalence in Caucasians. In total, in this study 6.84% of the studied patients carried pathogenic variants in *LRRK2*. Therefore, our results provide a very important role for *LRRK2* in typical EOPD in our Spanish population. This is according with recent studies in other populations, such a large UK population based study where it has been shown that *LRRK2* mutations are present at a significant rate in patients under 50 years [22].

Pathogenic variants in the *GIGYF2* gene have been related to familial PD [5], but some studies in diverse populations showed that there is no correlation between the presence of variants in this gene with the disease [23–25]. In this analysis, we found a patient (EP-33) with co-occurrence of c.4536+3A>G (predicted affecting splicing) in *LRRK2* and p.Arg1225Cys in *GIGYF2* pathogenic variants. This patient presented cognitive impairment, as previously described in a Spanish family featuring late-onset PD and cognitive impairment and with a genetic variant, in *GIGYF2*, identified as potential disease-causing variation [26]. It is interesting to note that both genes (*LRRK2* and *GIGYF2*) have been described as playing some roles in the autophagy process [27]. As such, this kind of co-occurrence involving both genes has been previously described and associated with an earlier age at onset of PD, suggesting additive effects [5]. Further genetic studies and functional analysis are necessary to conclude the implications in PD of variants located in *GIGYF2*.

In any case, although these two cases could indicate a modifying effect, this cannot be determinate in this study, as this is still on an exploratory level.

Among risk factors in PD, *GBA* is well known [28]. Variantss in this gene are present in multiple ethnicities [29]. Heterozygous pathogenic variants are dominantly inherited, and the penetrance is high, related to EOPD [30]. Importantly, the frequency of *GBA* pathogenic variants in our population is rather low. Indeed, our *GBA* mutational spectrum did not include the most frequent pathogenic variants L444P and N370S [31]. Technical reasons or variant filtering could explain this. Therefore, *GBA* sequencing using targeted next generation sequencing is difficult because the existence of the pseudo-*GBA*, with very similar sequence. In addition, it has been shown that *GBA* variants have a different impact on the PD phenotype according to their pathogenicity (both deleterious and benign). Because of preselecting for pathogenic, likely pathogenic or VOUS (variant of unknown significance) variants, it may very well be an underestimate of the true yield of variants in *GBA* affecting Parkinson’s disease in our population.

This study focused on exonic and intronic variants; however, most of the variants found were located in the untranslated regions (mainly in 3’UTR). Interestingly, heterozygous pathogenic variants in recessive genes were detected in some patients that also presented variants in untranslated regions. Therefore, it will be important to perform further deeper analysis in order to study their contribution to disease pathogenesis.

Finally, although Pool-seq is a cost-effective alternative to individual sequencing, there are some limitations to consider; in particular, the presence of false positives and/or false negatives. It has been described that the accuracy of Pool-seq increases with the number of individuals included in the pool and with a high sequencing depth [32, 33]. However, increasing sequencing depth could give rise to other problems, such as to confound sequencing errors with very rare alleles that could lead to an increase of the false positive rate. We think that our approach using, among others, a high number of individuals included in each pool, a high depth of coverage, and a threshold on the minimum percentage of reads of alternative alleles, minimizes the errors and produces data with satisfactory precision and accuracy.

## Conclusions

This study, in terms of cohort size, number of included genes and applied methods is the first systematic study of genetic variability in PD-related genes in EOPD patients from Spain. Our results provide a comprehensive genetics profile of EOPD. In addition, further evidence for gene interaction involving *ATP13A2* and *PARK2*, and *LRRK2* and *GIGYF2*, are provided with co-occurrence of pathogenic variations in both genes. Moreover, our study suggests a greater role for *LRRK2* in typical, idiopathic EOPD than previously believed in the Spanish population. Thus, our results could weight utility in personalized genetic counselling.

## Supporting information

S1 Methods (DOCX)

S1 Table Summary of deleteriousness prediction methods used in our study. (PDF)

S2 Table Description of 32 preselected candidate pathogenic variants after applying bioinformatics filters. Nonsense variants and those missense variants which were predicted as likely pathogenic by at least 53’9% (seven of thirteen) of the bioinformatics tools.
(PDF)

S3 Table Very interesting variants to be prioritized found in our PD-population from central Spain. (PDF)

S4 Table Variants putatively affecting splicing. (PDF)
