# Correction: A genetic analysis of a Spanish population with early onset Parkinson’s disease

**DOI:** 10.1371/journal.pone.0249805

**Published:** 2021-04-05

**Authors:** The PLOS ONE Staff

The first author’s name is listed incorrectly. The correct name is: Cristina Tejera-Parrado.

The second author’s name is listed incorrectly. The correct name is: Pablo Mir.

The third author’s name is listed incorrectly. The correct name is: María Teresa Periñán.

The fourth author’s name is listed incorrectly. The correct name is: Lydia Vela-Desojo.

The fifth author’s name is listed incorrectly. The correct name is: Irene Abreu-Rodríguez.

The sixth author’s name is listed incorrectly. The correct name is: Araceli Alonso-Cánovas.

The seventh author’s name is listed incorrectly. The correct name is: Inmaculada Bernal-Bernal.

The eighth author’s name is listed incorrectly. The correct name is: Marta Bonilla-Toribio.

The ninth author’s name is listed incorrectly. The correct name is: Dolores Buiza-Rueda.

The tenth author’s name is listed incorrectly. The correct name is: María José Catalán-Alonso.

The eleventh author’s name is listed incorrectly. The correct name is: Rocío García-Ramos.

The twelfth author’s name is listed incorrectly. The correct name is: Pedro José García-Ruiz.

The thirteenth author’s name is listed incorrectly. The correct name is: Ismael Huertas-Fernández.

The fourteenth author’s name is listed incorrectly. The correct name is: Silvia Jesús.

The fifteenth author’s name is listed incorrectly. The correct name is: Miguel A Labrador-Espinosa.

The sixteenth author’s name is listed incorrectly. The correct name is: Lydia López-Manzanares.

The seventeenth author’s name is listed incorrectly. The correct name is: Juan Carlos Martínez-Castrillo.

The nineteenth author’s name is listed incorrectly. The correct name is: Ana Rojo-Sebastián.

The twentieth author’s name is listed incorrectly. The correct name is: Cristina Ruiz-Huete.

The twenty-first author’s name is listed incorrectly. The correct name is: Javier Del Val.

The twenty-second and corresponding author’s name is listed incorrectly. The correct name is: Pilar Gómez-Garre P.

The correct citation is: Tejera-Parrado C, Mir P, Periñán MT, Vela-Desojo L, Abreu-Rodríguez I, Alonso-Cánovas A, et al. (2020) A genetic analysis of a Spanish population with early onset Parkinson’s disease. PLoS ONE 15(9): e0238098. https://doi.org/10.1371/journal.pone.0238098.

The publisher apologizes for the errors.
