# Supplementary material for: A genetic analysis of a Spanish population with early onset Parkinson’s disease
Source: PLoS One. 2020 Sep 1;15(9):e0238098. doi: 10.1371/journal.pone.0238098 (PMC7462269; doi:10.1371/journal.pone.0238098)
Supplement: S1 Methods — (DOCX) [file pone.0238098.s001.docx]

**METHODS S1**

***Ethics Statements***

The study was approved by the CEIs (Comités de Ética en Investigación) from all participating centers:
Comité de Ética de la Investigación del Hospital Universitario Virgen del Rocío
Comité Ético de Investigación Clínica del Hospital Universitario Fundación Alcorcón
Comité de Ética de la Investigación del Hospital Universitario Ramón y Cajal
Comité Ético de Investigación Clínica del Hospital Clínico San Carlos
Comité de Ética de la Investigación Clínica de la Fundación Jiménez Díaz
Comité Ético de Investigación Clínica del Hospital Universitario 12 de Octubre
Comité Ético de Investigación Clínica del Hospital Universitario de la Princesa
Comité de Ética de la Investigación del Hospital Universitario Príncipe de Asturias
In addition, the study was conducted according to the principles expressed in the Helsinki Declaration. Each individual who participated in the study signed a written informed consent form prior to blood withdrawal

**Prediction of the significance of variants**

For the prediction of the significance of variants we followed the combination of several criteria:

1) Type of the variation: whether variation results in a null variant: that is nonsense, frameshift or affecting to canonical splice sites that result in frameshift of the coding sequence, mutations affecting the initiation codon, and those leading to exon(s) deletion.

2) In-silico prediction: We used multiple bioinformatics tools and the impact was interpreted with respect to all known isoforms of each gene. For missense variants, a total of thirteen tools were examined. Therefore, we used 6 functional prediction scores (SIFT, Polyphen-2, MutationTaster, MutationAssesor, LRT, and FATHMM), 4 conservation scores (GERP++, PhyloP, PhastCons, and Grantham Score), and three ensemble-based prediction scores (CADD, LR, and RadialSVM). A threshold was set for each tool above in which the variant was classified as damaging (Table S1). In addition, all variants were analyzed using GWAVA, CADD, and the splicing predictions from the Batch version of the annotation software package Alamut (Interactive Biosoftware, Rouen, France), that integrates 5 sources: Splice Site Finder, MaxEntScan, NNSplice, GeneSplicer and Human Splicing Finder (Table S1). We also considered the dbscsnv11 database for predicting the splicing impact by Ada Boost and Random Forest (Table S1). Moreover, non-coding variants were prioritized when ReMM-score >0.8 and IW-score with an associated *P*-value<0.05.

3) Score from Regulome-DB of 1.

4) Co-occurrence with pathogenic variants.
