# Supplementary material for: A genetic analysis of a Spanish population with early onset Parkinson’s disease
Source: PLoS One. 2020 Sep 1;15(9):e0238098. doi: 10.1371/journal.pone.0238098 (PMC7462269; doi:10.1371/journal.pone.0238098)
Supplement: S1 Table — (PDF) [file pone.0238098.s002.pdf]

**Table S1.** Summary of deleteriousness prediction methods used in our study.

| Name | Category | Thereshold |
|---|---|---|
| SIFT | Function prediction | < 0.05 |
| Polyphen-2 | Function prediction | > 0.85 |
| Mutation Taster | Function prediction | Disease causing o Disease causing automatic |
| PhyloP | Conservation score | > 0.7 |
| CADD | Ensembl score | > 10 |
| Grantham Score | Conservation score | > 100 |
| PhastCons | Conservation score | > 0.7 |
| GERP++ | Conservation score | > 3 |
| LRT | Function prediction | Deleterious |
| Mutation Assessor | Function prediction | High o Medium |
| LR | Ensembl score | Deleterious |
| FATHMM | Function prediction | Deleterious |
| RadialSVM | Ensembl score | Deleterious |
| SSF | Effect on splicing | > 70 |
| MaxEnt | Effect on splicing | > 0 |
| NNSPLICE | Effect on splicing | > 0.4 |
| GeneSplicer | Effect on splicing | > 0 |
| HSF | Effect on splicing | > 65 |
| dbscSNV_RF_Score | Effect on splicing | > 0.6 |
| dbscSNV_Ada_Score | Effect on splicing | > 0.6 |
