# Supplementary material for: A genetic analysis of a Spanish population with early onset Parkinson’s disease
Source: PLoS One. 2020 Sep 1;15(9):e0238098. doi: 10.1371/journal.pone.0238098 (PMC7462269; doi:10.1371/journal.pone.0238098)
Supplement: S2 Table — Nonsense variants and those missense variants which were predicted as likely pathogenic by at least 53’9% (seven of thirteen) of the bioinformatics tools. (PDF) [file pone.0238098.s003.pdf]

Table S2. Description of 32 preselected candidate pathogenic variants after applying bioinformatics filters. Nonsense variants and those missense variants which were predicted as likely pathogenic by at least 53’9% (seven of thirteen) of the bioinformatics tools.

Gene	Chr: position	Amino acid change	Nucleotide change	Zygosity	SIFT		Polyphen-2		MutationTaster		PhyloP	CADD	Grantham Score	Phast Cons	GERP++	MutationAssessor		LRT	FATHMM	RadialSVM	LR	ClinVar	N
					Score	Prediction	Score	Prediction	Score	Prediction						Score	Prediction						
ATP13A2	1:17328585	p.G217S	c.G649A	Hetero	0	Damaging	1	Probably damaging	1	Disease causing	5.577	22.8	56	0.956	4.89	3.41	Medium	Deleterious	Deleterious	Deleterious	Deleterious	VOUS	1
ATP13A2	1:17318246	p.R745H	c.G2234A	Hetero	0.03	Damaging	1	Probably damaging	1	Disease causing	7.265	21.1	29	1	4.93	2.315	Medium	Deleterious	Tolerated	Deleterious	Deleterious	ND	1
DNAJC6	1:65831737	p.S134F	c.C401T	Hetero	0.09	Tolerated	1	Probably damaging	1	Disease causing	7.247	29.1	155	1	5.51	2.62	Medium	Deleterious	Deleterious	Deleterious	Deleterious	ND	1
DNAJC6	1:65854097	p.K398E	c.A1192G	Hetero	0	Damaging	0.992	Probably damaging	1	Disease causing	6.548	20.4	56	1	4.9	2.44	Medium	Deleterious	Deleterious	Deleterious	Deleterious	ND	1
DNAJC6	1:65877062	p.V922M	c.G2764A	Hetero	0	Damaging	1	Probably damaging	1	Disease causing	9.657	29.6	21	1	5.03	3.03	Medium	Deleterious	Tolerated	Tolerated	Tolerated	ND	1
EIF4G1	3:184040997	p.G693C	c.G2077T	Hetero	0.05	Damaging	0.999	Probably damaging	1	Disease causing	0.872	18.54	159	1	5.16	0.895	Low	Deleterious	Tolerated	Tolerated	Tolerated	ND	1
EIF4G1	3:184045661	p.L1282H	c.T3845A	Hetero	0.56	Tolerated	1	Probably damaging	1	Disease causing	7.698	31	99	1	6.17	1.74	Low	Deleterious	Tolerated	Tolerated	Tolerated	ND	2
EIF4G1	3:184046529	p.M1362T	c.T4085C	Hetero	0	Damaging	0.998	Probably damaging	1	Disease causing	7.698	18.64	81	1	4.54	2.48	Medium	Deleterious	Tolerated	Tolerated	Tolerated	ND	1
GBA	1:155207997	p.V230G	c.T689G	Hetero	0.25	Tolerated	0.011	Benign	1	Disease causing	5.309	4.868	109	0.987	2.5	0.77	Non-functional	Neutral	Deleterious	Deleterious	Deleterious	ND	1
GBA	1:155206220	p.I347T	c.T1040C	Hetero	0.11	Tolerated	0.937	Possibly damaging	1	Disease causing	8.274	17.27	89	0.984	3.67	1.365	Low	Deleterious	Deleterious	Deleterious	Deleterious	ND	1
GBA	1:155206167	p.E365K	c.G1093A	Hetero	0.43	Tolerated	0.03	Benign	0	Disease causing	0.838	11.06	56	0.845	3.67	1.01	Low	Neutral	Deleterious	Deleterious	Deleterious	VOUS / RF	1
GBA	1:155206052	p.S403N	c.G1208A	Hetero	0.1	Tolerated	0.996	Probably damaging	0.554	Polymorphism	5.361	20.3	46	1	3.57	2.045	Medium	Deleterious	Deleterious	Deleterious	Deleterious	P	1
GIGYF2	2:233712207	p.R1225C	c.C3673T	Hetero	0	Damaging	0.946	Possibly damaging	1	Disease causing	1.725	17.35	180	0.997	5.4	0.625	Non-functional	Deleterious	Tolerated	Tolerated	Tolerated	ND	1
LRRK2	12:40704236	p.R1441C	c.C4321T	Hetero	0.14	Tolerated	1	Probably damaging	1	Disease causing	3.654	23.5	180	0.082	4.74	1.97	Medium	Deleterious	Tolerated	Deleterious	Deleterious	P	1
LRRK2	12:40713845	p.R1628H	c.G4883A	Hetero	0.22	Tolerated	1	Probably damaging	1	Disease causing	8.853	33	29	1	5.54	1.43	Low	Deleterious	Tolerated	Tolerated	Tolerated	VOUS	2
LRRK2	12:40734202	p.G2019S	c.G6055A	Hetero	0	Damaging	1	Probably damaging	1	Disease causing	9.527	36	56	0.966	5.69	3.765	High	Deleterious	Deleterious	Deleterious	Deleterious	P	5
PARK2	6:162864434	p.K27X	c.A79T	Homo	1	Tolerated	.	.	1	Disease causing	6.231	38	NA	1	5.63	.	.	Deleterious	.	.	.	ND	1
PARK2	6:162864388	p.R42H	c.G125A	Hetero	0.15	Tolerated	0.726	Possibly damaging	1	Disease causing	0.727	21.2	103.29	1	4.74	3.47	Medium	Deleterious	Tolerated	Deleterious	Deleterious	VOUS	1
PARK2	6:162394349	p.T240M	c.C719T	Hetero	0.05	Damaging	0.991	Probably damaging	0.983	Polymorphism	6.594	16.31	81	0.299	5.14	1.78	Low	Neutral	Deleterious	Deleterious	Deleterious	P	1
PARK2	6:162206909	p.R256C	c.C766T	Hetero	0.02	Damaging	1	Probably damaging	0.999	Disease causing	8.289	26.9	180	0.06	5.75	2.3	Medium	Deleterious	Deleterious	Deleterious	Deleterious	ND	1
PARK2	6:161781200	p.R402H	c.G1205A	Hetero	0.01	Damaging	1	Probably damaging	1	Disease causing	5.469	21.2	29	0.686	4.89	2.56	Medium	Deleterious	Deleterious	Deleterious	Deleterious	ND	1
PARK2	6:161771195	p.W445X	c.G1334A	Hetero	1	Tolerated	.	.	1	Disease causing	5.476	34	NA	1	5.27	.	.	Deleterious	.	.	.	ND	1
PINK1	1:20972133	p.L347P	c.T1040C	Homo	0	Damaging	1	Probably damaging	1	Disease causing	6.959	15.71	98	0.775	4.9	4.13	High	Deleterious	Tolerated	Deleterious	Deleterious	P	1
PLA2G6	22:38509628	p.V690I	c.G2068A	Hetero	0	Damaging	1	Probably damaging	1	Disease causing	4	17.29	29	0.962	4.36	1.85	Low	Deleterious	Tolerated	Deleterious	Deleterious	VOUS	1
PLA2G6	22:38508553	p.R745Q	c.G2234A	Hetero	0	Damaging	1	Probably damaging	1	Disease causing	6.995	28.7	43	1	4.54	2.64	Medium	Deleterious	Tolerated	Deleterious	Deleterious	ND	1
SMPD1	11:6414486	p.R378C	c.C1132T	Hetero	0	Damaging	1	Probably damaging	1	Disease causing	4.64	22.4	180	1	5.41	3.785	High	Deleterious	Deleterious	Deleterious	Deleterious	ND	1
SMPD1	11:6415566	p.R542Q	c.G1625A	Hetero	0.14	Tolerated	0.981	Probably damaging	0.75	Disease causing	1.982	13.8	43	0.919	2.94	2.255	Medium	Neutral	Deleterious	Deleterious	Tolerated	VOUS	1
SYNJ1	21:34060664	p.R307H	c.G920A	Hetero	0.64	Tolerated	1	Probably damaging	1	Disease causing	7.193	18.67	29	1	6.16	1.145	Low	Deleterious	Tolerated	Tolerated	Tolerated	VOUS	1
SYNJ1	21:34060625	p.A320V	c.C959T	Hetero	0.02	Damaging	1	Probably damaging	1	Disease causing	9.061	36	64	1	6.16	2.11	Medium	Deleterious	Tolerated	Tolerated	Tolerated	VOUS	1
SYNJ1	21:34003786	p.G1453E	c.G4358A	Hetero	0.01	Damaging	0.989	Probably damaging	1	Disease causing	4.989	22.9	NA	0.988	5.13	1.845	Low	Deleterious	Tolerated	Tolerated	Tolerated	B	4
VPS35	16:46717460	p.Q21L	c.A62T	Hetero	0.32	Tolerated	0	Benign	1	Disease causing	7.972	15.92	113	1	5.42	0.64	Non-functional	Deleterious	Tolerated	Tolerated	Tolerated	ND	2
VPS35	16:46696262	p.A654S	c.G1960T	Hetero	0.08	Tolerated	0.999	Probably damaging	1	Disease causing	7.818	34	99	1	5.49	2.87	Medium	Deleterious	Tolerated	Deleterious	Deleterious	ND	1

VOUS: Variant of uncertain significance; P: Pathogenic; B: Benign; N: number of carriers; ND: No data.
