# Supplementary material for: A genetic analysis of a Spanish population with early onset Parkinson’s disease
Source: PLoS One. 2020 Sep 1;15(9):e0238098. doi: 10.1371/journal.pone.0238098 (PMC7462269; doi:10.1371/journal.pone.0238098)
Supplement: S3 Table — (PDF) [file pone.0238098.s004.pdf]

Table S3. Very interesting variants to be prioritized found in our PD-population from central Spain

Gene (MI)	Transcript	Exon (E) / Intron (I)	Nucleotide change	Amino acid change	dbSNP ID	Carriers (n=117)		maf			Previously Reported	Effect	ACMG classification (AC)	
						Het	Hom	Our study	GO-ESP[&]	ExAc*			Verdict	Rules
ATP13A2 **(AR-RF)**	NM_022089.3	E8	c.649G>A	p.Gly217Ser	rs199961048	1	0	4.27e-3	ND	1.62e-05	Yes	MS	VOUS	PM2, PP3, BP1
		E20	c.2234G>A	p.Arg745His	rs140455242	1	0	4.27e-3	1.16e-04	1.5e-05	Yes	MS	VOUS	PM2, PP3, BP1
		I24	c.2762+21T>C	-	rs560897844	1	0	4.27e-3	ND	1.5e-05	Yes	SR	VOUS	PM2, PP3, BP4
DNAJC6 **(AR)**	NM_001256864	E4	c.401C>T	p.Ser134Phe	rs1226853857	1	0	4.27e-3	ND	ND	Yes	MS	VOUS	PM2, PP3, BP1
		E9	c.1192A>G	p.Lys398Glu	-	1	0	4.27e-3	ND	ND	No	MS	VOUS	PM2, PP3, BP1
		E18	c.2764G>A	p.Val922Met	-	1	0	4.27e-3	ND	ND	No	MS	VOUS	PM2, PP3, BP1
EIF4G1 **(AD)**	NM_001194946	E15	c.2077G>T	p.GLy693Cys	rs112019125	1	0	4.27e-3	ND	9e-05	Yes	MS/SR	LB	PP3, BS1, BP1
		E28	c.4085T>C	p.Met1362Thr	rs144059151	1	0	4.27e-3	8.14e-04	5.8e-04	Yes	MS	LB	PP3, BS1, BP1
GBA **(AD-RF)**	NM_001005741.2	E9	c.1040T>C	p.Ile347Thr	-	1	0	4.27e-3	ND	ND	No	MS	LP	PM1, PM2, PP2, PP3
		E9	c.1208G>A	p.Ser403Asn	-	1	0	4.27e-3	ND	ND	No	MS	LP	PM1, PM2, PM5, PP2, PP3
GIGYF2 **(AD-S)**	NM_001103147	E29	c.3673C>T	p.Arg1225Cys	rs149440697	1	0	4.27e-3	1.16e-04	4.47e-05	Yes	MS	VOUS	PP3, BS1
LRRK2 **(AD)**	NM_198578.3	E30	c.4229C>T	p.Thr1410Met	rs72546327	1	0	4.27e-3	0	1.2e-04	Yes	MS	B	PM1, PP3, BS1, BS2, BP1, BP6
		E31	c.4321C>T	p.Arg1441Cys	rs33939927	1	0	4.27e-3	ND	1.5e-05	Yes	MS	LP	PM1, PM2, PM5, PP3, PP5, BP1
		I31	c.4536+3A>G	-	rs41286476	1	0	4.27e-3	5.81e-04	4.38e-04	Yes	SR	VOUS	PP3, PP5, BS2
		E41	c.6055G>A	p.Gly2019Ser	rs34637584	5	0	0.022	5.8e-04	6.29e-04	Yes	MS	P	PS1, PS3, PS4, PM1, PP3, BS2,
PARK2 **(AR)**	NM_004562.2	2	c.79A>T	p.Lys27Stop	-	0	1	8.55e-3	ND	ND	No	SG	P	PVS1, PM1, PM2, PP3
		2	c.125G>C	p.Arg42Pro	rs368134308	1	0	4.27e-3	1.16e-04	4.5e-05	Yes	MS	LP	PM1, PM2, PP2, PP3, PP5
		2	c.155delA	p.Asn52Metfs	rs754809877	3	7	0.073	6.06e-04	3.15e-04	Yes	FS	P	PVS1, PM1, PP3, PP5
		6	c.719C>T	p.Thr240Met	rs137853054	1	0	4.27e-3	2.33e-04	1.35e-04	Yes	MS	LP	PM1, PM5, PP2, PP3, PP5
		7	c.766C>T	p.Arg256Cys	rs150562946	1	0	4.27e-3	4.65e-04	5.14e-04	Yes	MS	VOUS	PM1, PP2, PP3, PP5
		11	c.1205G>A	p.Arg402His	rs766915327	1	0	4.27e-3	ND	0	Yes	MS	LP	PM1, PM2, PM5, PP2, PP3
		12	c.1334G>A	p.Trp445Stop	rs961239925	1	0	4.27e-3	ND	ND	Yes	SG	P	PVS1, PM1, PM2, PP3
		3	Del	Del	-	0	1	8.55e-3	-	-	-	DEE	P	PVS1, PS3

		4	Del	Del	-	1	0	4.27e-3	-	-	-	DEE	LP	PVS1, PM3
		3, 4, 5, 6	Del	Del	-	1	2	0.021	-	-	-	DEE	LP	PVS1, PM3
		7	Del	Del	-	4	0	0.017	-	-	-	DEE	LP	PVS1, PM3
***PINK1* (AR)**	NM_032409.2	5	c.1040T>C	p.Leu347Pro	rs28940285	0	1	8.55e-3	ND	0	Yes	MS	LP	PM1, PM2, PP3, PP5, BP1
***PLA2G6* (AR)**	NM_003560.2	E15	c.2068G>A	p.Val690Ile	rs141777179	1	0	4.27e-3	8.14e-04	9.17e-04	Yes	MS	VOUS	PP2, PP3, BS1
		E16	c.2234G>A	p.R745Q	rs368514303	1	0	4.27e-3	1.2e-04	0	Yes	MS	LP	PM1, PM5, PP2, PP3, BS1
***SMPD1* (RF)**	NM_000543.4	E2	c.441G>A	p.V147V	rs148944108	2	0	8.55e-3	0	1.67e-04	Yes	SN / SR	VOUS	PM1, PP3, BS1
		E3	c.1132C>T	p.R378C	rs369088417	1	0	4.27e-3	0	0	Yes	MS	LP	PM1, PM2, PP2, PP3
***SYNJ1* (AR)**	NM_003895.3	E7	c.959C>T	p.A320V	rs1046699971	1	0	4.27e-3	ND	ND	Yes	MS	VOUS	PM2, PP3, BP1
***VPS35* (AD)**	NM_018206.5	E15	c.1960G>T	p.A654S	-	1	0	4.27e-3	No	ND	No	MS	VOUS	PM2, PP3, BP1

MI: Mode of Inheritance; AD: Autosomal dominant; AR: Autosomal recessive; S: Susceptibility; RF: Risk factor; N: Number of carriers; Het: Heterozygosity; Hom: Homozygosity; SG: Stop gain; MS: Missense; FS: Frameshift; DEE: Deletion of the entire exon; SR: Splice region; SN: Synonymous; MAF: Minor allele frequency; &: European Americans; *: Non-Finnish European; AC: Automated criteria; VOUS: Variant of uncertain significance; LP: Likely Pathogenic; P: Pathogenic; LB: Likely Benign; B: Benign.
