# Supplementary material for: A genetic analysis of a Spanish population with early onset Parkinson’s disease
Source: PLoS One. 2020 Sep 1;15(9):e0238098. doi: 10.1371/journal.pone.0238098 (PMC7462269; doi:10.1371/journal.pone.0238098)
Supplement: S4 Table — (PDF) [file pone.0238098.s005.pdf]

online only

Table S4. Variants putatively affecting splicing

Gene	Intron / Exon	Chr position	Nucleotide change	Strand	Splice Site	Distance to Splice Site	ID	N	Frequency		SSF		MaxEnt		NNSplice		GeneSplicer		HSF		dncsSNV scores		Putative consequence
									1000G EUR	Affected	Score	variation	Score	variation	Score	variation	Score	variation	Score	variation	ada	rf	
ATP13A2	E10	1: 17326767	c.881G>A	-	5'	27	rs56367069*	5	0.018	0.0214	79.69	(+12.7%)	6.46	(+55.9%)	0.79	appear	6.76	(+142%)	87.86	(+5.9%)	-	-	appear a putative new 5' donor site in c.882
	I24	1: 17314796	c.2762+21T>C	-	5'	19	rs560897844*	1	0.0002 (all)	0.0043	81.66	(+5.3%)	8.48	(+25.7%)	0.54	appear	5.18	(+146.3%)	92.05	(+2.2%)	-	-	stronger criptic 5' donor site
	I26	1: 17313454	c.3084-3C>T	-	3'	1	rs7531163	68	0.25	0.325	76.52	(-7.5%)	9.90	(+6.5%)	0.96	(+4.4%)	13.19	(+14.3%)	84.49	(-8.3%)	0.0011	0.1	weaker 3' acceptor site
	I27	1: 17313283	c.3235+17G>A	-	5'	15	rs56146840	5	0.019	0.0214	70.14	appear	3.78	appear	-	-	1.96	appear	83.47	appear	-	-	appear a new 3' acceptor site in c.3235+18
DNAJC6	E9	1: 65854097	c.1192A>G	+	5'	2	-	1	-	0.0043	78.90	(-10.2)	10.15	(+0.7%)	0.99	(-0.2%)	4.94	(+21.9%)	84.56	(-5.4%)	0.9934	0.96	weaker 5' donor site in c.1193
EIF4G1	I11	3: 184040606	c.1796-3C>T	+	3'	1	rs190378563	1	0.001	0.0043	87.71	(-6.6%)	11.84	(-12.84%)	0.99	(+0.2%)	10.07	(-12.9%)	87.91	(-8.0%)	0.0018	0.196	weaker 3' acceptor site
	E13	3: 184040997	c.2056G>T	+	5'	33	rs112019125*	1	-	0.0043	80.14	appear	5.28	appear	0.92	appear	2.52	appear	87.15	appear	-	-	appear a putative new 5' donor site in c.2054
	I29	3: 184049399	c.4398+5G>A	+	5'	3	rs16858676	1	0.014 (all)	0.0043	74.71	(-14.0%)	6.04	(-37.4%)	0.88	(-9.3%)	6.17	(-37.3%)	77.73	(-13.5%)	0.9556	0.656	weaker 5' donor site
GIGYF2	I4	2: 233568203	c.-44+4A>G	+	5'	2	rs746723892	1	-	0.0043	89.92	(-10.1%)	10.28	(-5.3%)	0.96	(-3.4%)	5.73%	(-16.2%)	91.66	(-8.3%)	0.3339	0.44	weaker 5' donor site
LRRK2	I31	12: 40704454	c.4536+3A>G	+	5'	1	rs41286476	1	-	0.0043	77.31	(-5.3%)	5.19	(-41.8%)	desappear	-	desappear	-	86.21	(-11.4%)	0.9712	0.86	weaker 5' donor site
SMPD1	E2	11: 6412736	c.441G>A	+	3'	120	rs148944108*	2	0.002	0.0086	81.75	(+5.6%)	8.98	(+100.6%)	0.75	appear	4.85	appear	87.50	(+1.3%)	-	-	appear a new 5' donor site in c.438
SYNJ1	I1	21: 34100124	c.-23+24G>A	-	5'	22	rs755252765	2	-	0.0086	70.57	appear	5.10	(+79.3%)	-	-	4.73	(+60.9%)	82.29	(+1.0%)	-	-	appear a putative new 5' donor site (refered to isoforms 3 and 4)
UCHL1	I2	41259149	c.45+6T>C	+	5'	4	rs11556273	21	0.09	0.098	77.42	(-6.9%)	5.89	(-29.9%)	0.90	(-8.3%)	4.22	(-34.4%)	85.13	(-2.5%)	0.8166	0.724	weaker 5' donor site

Chr: chromosome; E: Exon; I: Intron; N: number of carriers; SSF: Splicing Sequences Finder; MaxEnt: Maximum Entropy Scan; NNSplice: Splice site prediction by Neural Network: HSF: Human Splicing Finder; ADA: adaptive boosting, RF: random forest; *: Predicted as likely pathogenic by all 5 tools with high scores.
